# Mapping the output of the global literature on the links between gut microbiota and COVID-19

**DOI:** 10.1186/s41043-023-00346-w

**Published:** 2023-01-18

**Authors:** Sa’ed H. Zyoud, Muna Shakhshir, Amani S. Abushanab, Amer Koni, Moyad Shahwan, Ammar A. Jairoun, Samah W. Al-Jabi

**Affiliations:** 1 Poison Control and Drug Information Center (PCDIC), College of Medicine and Health Sciences, An-Najah National University, Nablus, 44839 Palestine; 2 Department of Clinical and Community Pharmacy, College of Medicine and Health Sciences, An-Najah National University, Nablus, 44839 Palestine; 3 Clinical Research Centre, An-Najah National University Hospital, Nablus, 44839 Palestine; 4 Department of Nutrition, An-Najah National University Hospital, Nablus, 44839 Palestine; 5 Division of Clinical Pharmacy, Hematology and Oncology Pharmacy Department, An-Najah National University Hospital, Nablus, 44839 Palestine; 6 College of Pharmacy and Health Sciences, Ajman University, Ajman, United Arab Emirates; 7 Health and Safety Department, Dubai Municipality, Dubai, United Arab Emirates

**Keywords:** COVID-19, Microbiota, Microbiome, Bibliometric

## Abstract

**Background:**

The term “human microbiota” refers to populations of microorganisms that live harmoniously in co-existence with humans. They contribute significantly to the host's immunological response when confronted with a respiratory viral infection. However, little is known about the relationship between the human microbiome and COVID-19. Therefore, our objective is to perform a bibliometric analysis to explore the overall structure and hotspots of research activity on the links between microbiota and COVID-19 at the global level.

**Methods:**

The research literature on the microbiota and COVID-19 published between 2020 and 2022 was obtained from the Scopus database. Bibliometric analysis and network visualization were performed with VOSviewer.

**Results:**

Of the 701 publications selected, the USA contributed the most (*n* = 157, 22.40%), followed by China (*n* = 118, 16.83%) and Italy (*n* = 82, 11.70%). Hotspots in this field were “COVID-19 is associated with an altered upper respiratory tract microbiome,” “the effect of antibiotics on the gut microbiome,” as well as “patient nutrition and probiotic therapy in COVID-19.”

**Conclusions:**

The links between microbiota and COVID-19 remain an urgent concern at present, and the use of probiotics or/and antibiotics during the pandemic needs to be further improved. This landscape analysis of the links between the microbiota and COVID-19 will provide a basis for future research.

## Introduction

Coronavirus disease (COVID-19) is caused by an infectious pathogen known as severe acute respiratory syndrome coronavirus 2 (SARS-CoV-2) [1]. This kind of virus affects the respiratory tract and causes various symptoms with different levels of consequences, from mild to serious or fatal clinical manifestations [2]. However, the pathogenicity of COVID-19 is not limited exclusively to the lungs. The gastrointestinal tract (GIT) is also affected by SARS-CoV-2 as a target [3, 4]. The healthy human gut microbiota consists of almost a trillion of microorganisms [5]. These microbes play a key role in the body through their metabolic, developmental, and protective action [6] that help in food digestion and impart protective systemic and pulmonary immunity. Once these protective barriers are damaged, microorganisms may translocate to other body sites like the respiratory tract and can eventually induce acute respiratory distress syndrome [7, 8].

Because the COVID-19 pandemic is still developing and is not completely under control, ongoing research is required to update current practices in light of this disease. Due to this, we thought it was important to perform a comprehensive and meticulous bibliometric review to provide an overview of research activity on the links between the microbiota and COVID-19 at the global level. Meanwhile, we identified the evolution of research hotspots and forecasted future research focuses using term clustering analysis, so supplying important data for follow-up investigations.

## Methods

We searched the Scopus database for documents for this study, as it has access to a large number of papers and provides more citation-rich. More than 34,000 peer-reviewed academic journals can be found in Scopus, the world's largest and most comprehensive academic information source. First, we searched for terms associated with COVID-19 in article titles, abstracts, and author keywords [9–11]. The search was then restricted to publications using phrases relating to the microbiota [12–14]. A publication date range of January 2020 to September 2022 was chosen to restrict the investigation further. On September 30, 2022, the data were downloaded. Bibliometric analysis was performed with VOSviewer (version 1.6.18), and the results were visualized with the use of network maps. This resulted in improved subject comprehension and interpretation [15]. This article identified the research lines, the countries with the highest number of publications, the study areas, and the articles with the highest number of citations.

## Results

The publications on COVID-19 and microbiota analyzed in this study were 701, of which 107 were published in 2020, 249 in 2021, and 245 in 2022. Of all these publications, 334 (47.65%) were articles, 249 (35.52%) were reviews, and 118 (16.83%) were others (e.g., letters, editorials). Research on the links between COVID-19 and microbiota has been paid more and more attention. As a result, the volume of annual publications has grown over time. In addition, during the coming years, it is anticipated that the annual publication output is anticipated to expand rapidly, indicating a promising future for this area of study.

**Fig. 1:**
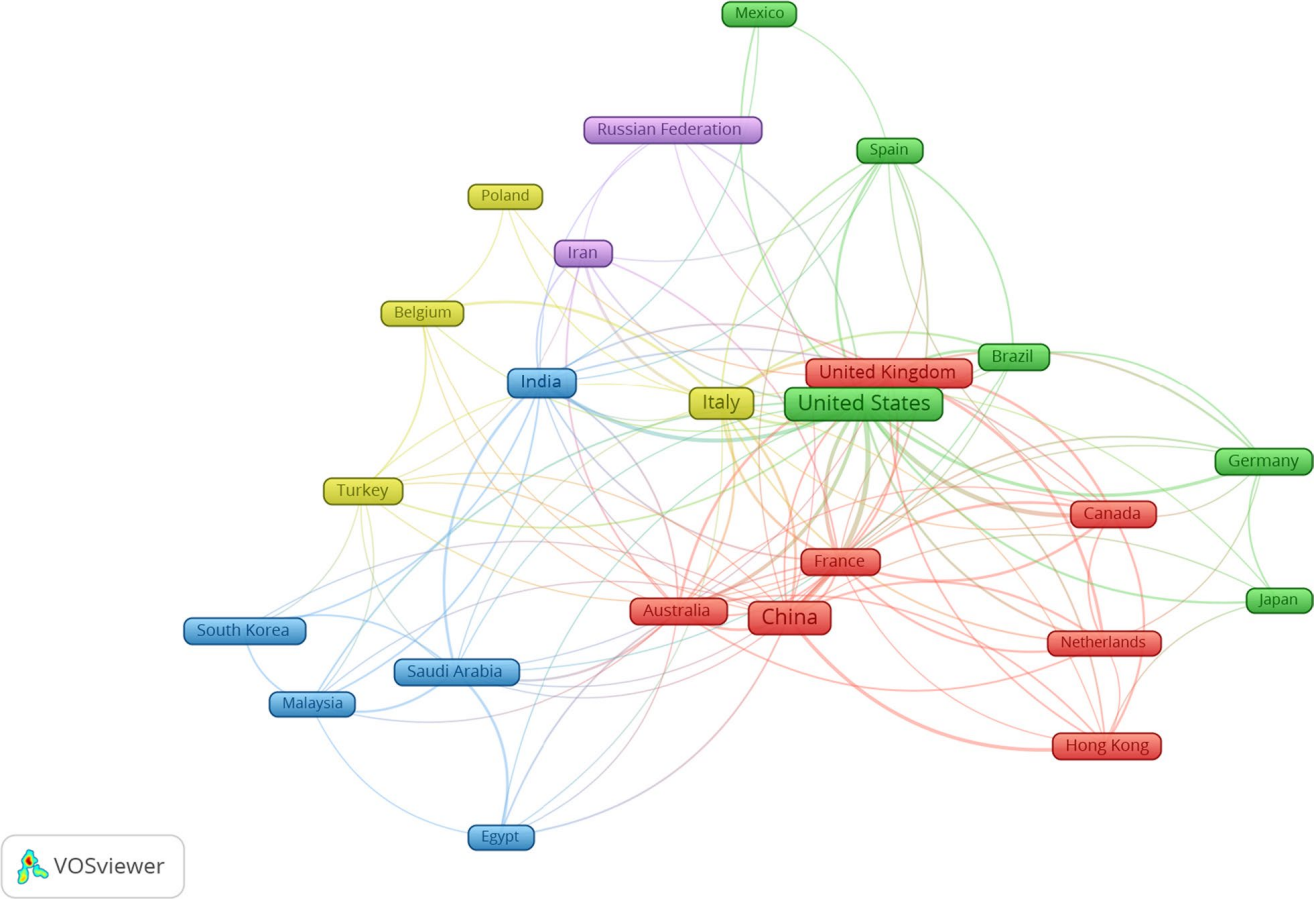
Collaborative relationship between different countries

The included studies were conducted in 97 countries of the world. The top 10 publications on the links between COVID-19 and microbiota were the USA (*n* = 157, 22.40%), China (*n* = 118, 16.83%), Italy (*n* = 82, 11.70%), India (*n* = 61, 8.71%), UK (*n* = 42, 5.99%), Iran (*n* = 32, 4.56%), Australia (*n* = 28, 3.99%), Brazil (*n* = 28, 3.99%), Germany (*n* = 25, 3.57%), and France (*n* = 24, 3.42%). A map showing international cooperation between countries and regions shows that the USA and China collaborate with the majority of countries in publication (Fig. 1).

The ten most cited papers received 2,446 citations in all [16–25]. Total citations of these articles that cited the connection between microbiota and COVID-19 research ranged from 102 to 580 (Table 1).

**Table 1:** Ten most cited publications in research related to microbiota and COVID-19

Authors	Title	Year	Source title	Cited by
Zuo et al. [25]	“Alterations in Gut Microbiota of Patients With COVID-19 During Time of Hospitalization”	2020	Gastroenterology	580
Yeoh et al. [22]	“Gut microbiota composition reflects disease severity and dysfunctional immune responses in patients with COVID-19”	2021	Gut	334
Dhar and Mohanty [17]	“Gut microbiota and Covid-19—possible link and implications”	2020	Virus Research	313
Gu et al. [18]	“Alterations of the gut microbiota in patients with coronavirus disease 2019 or H1N1 influenza”	2020	Clinical Infectious Diseases	296
Zuo et al. [23]	“Depicting SARS-CoV-2 fecal viral activity in association with gut microbiota composition in patients with COVID-19”	2021	Gut	196
Zuo et al. [24]	“Alterations in Fecal Fungal Microbiome of Patients With COVID-19 During Time of Hospitalization until Discharge”	2020	Gastroenterology	137
Saleh et al. [20]	“Mitochondria and microbiota dysfunction in COVID-19 pathogenesis”	2020	Mitochondrion	134
Villapol [21]	“Gastrointestinal symptoms associated with COVID-19: impact on the gut microbiome”	2020	Translational Research	133
Baud et al. [16]	“Using Probiotics to Flatten the Curve of Coronavirus Disease COVID-2019 Pandemic”	2020	Frontiers in Public Health	114
Infusino et al. [19]	Diet supplementation, probiotics, and nutraceuticals in SARS-CoV-2 infection: A scoping review	2020	Nutrients	107
Mak et al. [67]	“Probiotics and COVID-19: one size does not fit all”	2020	The Lancet Gastroenterology and Hepatology	102

**Fig. 2:**
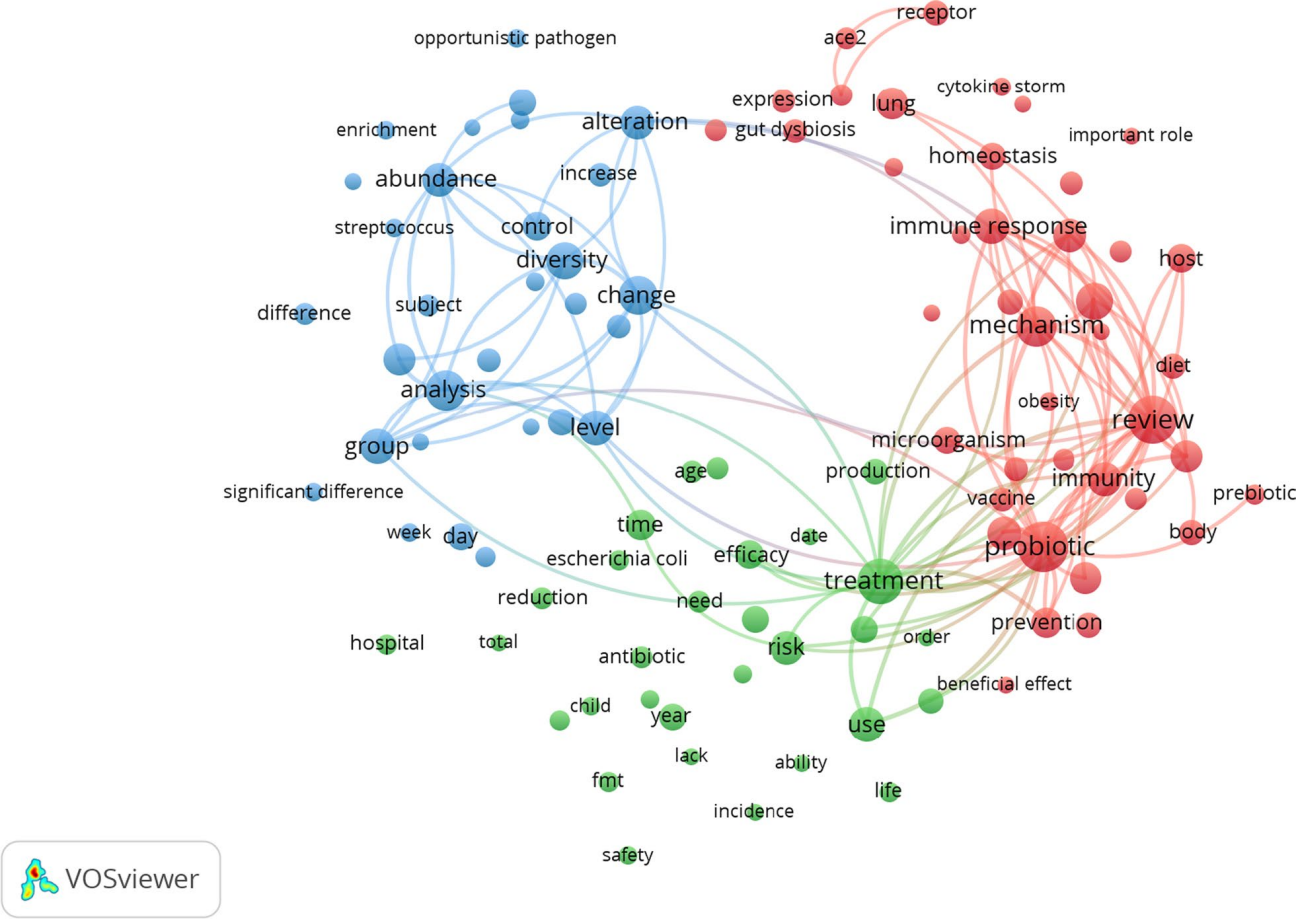
Analysis of research hotspots using cluster mapping. The VOSviewer software was used to evaluate words that appeared at least 20 times in titles and abstracts. The larger the circle of the keyword, the more frequently it appeared. The terms were classified into three clusters: “COVID-19 is associated with an altered upper respiratory tract microbiome” (blue cluster); “the effect of antibiotics on the gut microbiome” (green cluster); and “patient nutrition and probiotic therapy in COVID-19” (red cluster)

We used VOSviewer software to analyze the terms extracted from the titles and abstracts of the 701 publications. These terms were considered significant if they appeared in a location 20 or more times (Fig. 2). Our goal was to gain a comprehensive understanding of the primary research focus of these publications. A total of 168 terms met the requirements, and they were grouped into three groups: “COVID-19 is associated with an altered upper respiratory tract microbiome (blue cluster)”; “the effect of antibiotics on the gut microbiome (green cluster)” and “patient nutrition and probiotic therapy in COVID-19 (red cluster).”

## Discussion

In this bibliometric analysis study, we found 701 documents on the connection between microbiota and COVID-19 research from 2020 to 2022 in the Scopus database. This bibliometric network analysis has produced a map of global microbiota-related research on COVID-19, demonstrating a rapid increase in COVID-19 research driven by countries with many cases of the disease. According to the frequency of their publications, the USA and China were the two most productive countries in this field [26].

The results of this study indicate that the top 10 most cited references, which mainly refer to the composition of the gut microbiota, reflect the severity of COVID-19 or the role of probiotics in managing COVID-19 (such as the prevention of COVID-19). In terms of frequency, the most cited paper was “Alterations in Gut Microbiota of Patients With COVID-19 During Time of Hospitalization,” which was published by Zuo et al. [25] in 2020 article was cited 580 times. The authors of this study found ongoing changes in the fecal microbiota throughout hospitalization compared to controls. Alterations in the fecal microbiota were associated with the severity of COVID-19. Strategies to modify the gut microbiota can reduce the severity of the disease. The fact that many hot topics were published during this time, exposing new theories and establishing new research fields [16–25] like “gut microbiota composition reflects COVID-19 severity” or the “role of probiotics in the management of COVID-19,” can also be used to explain the rise in publications on the microbiota and COVID-19. The relationship between gut microbiota and COVID-19 severity raises novel therapeutic and diagnostic ideas and guidelines to enrich the diet with probiotics and prebiotics food and supplements as one of the treatment and prevention strategies [19, 27–32].

“COVID-19 is associated with an altered upper respiratory tract microbiome” as a theme was among the main hot topics in the current study. Any change in the composition, function, or diversity of the gut microbiota (gut dysbiosis) has been shown to affect the individual immunity of many systems, including the lungs [33]. On the other hand, lung inflammation can induce intestinal dysbiosis, especially since the respiratory system has its microbiota that is affected by any infections [34, 35], which is confirmed by the reported cases of patients with respiratory infections who usually have GIT dysfunction [36]. SARS-CoV-2 was already found in many parts of GIT, including the rectum, esophagus, duodenum, stomach, and fecal samples [37–39]. These patients often suffer from a lack of appetite, abdominal cramps, diarrhea, nausea, and vomiting [40, 41]. Several studies have repeatedly detected SARS-CoV-2 in samples from the stool and anal swabs of patients with COVID-19, suggesting the target site of viral replication and activity [42–44]. These results, along with the fact that diarrhea is one of the main GIT symptoms that some patients have, point to a possible link between the GIT and respiratory tract, or mainly, between the lungs and the intestinal microbiota, which is known as the intestinal–lung axis [45, 46].

Another key topic of discussion in the current investigation was the impact of antibiotics on the gut microbiome. Dysbiosis of the gut microbiota has been associated with autoimmune diseases, infectious diseases, autoimmune diseases, and allergy disorders [47]. When pathogens are eradicated with antibiotic therapy, the typical commensal microbiota may be indiscriminately eliminated, resulting in dysbiosis of the microecosystem. Compared to healthy controls, antibiotic therapy in COVID-19 patients can significantly affect intestinal flora [48]. Antibiotic overuse may have increased during the COVID-19 pandemic due to people buying antibiotics online pharmacies rather than visiting doctors in hospitals, as previously mentioned. Since the COVID-19 epidemic, 79–96% of antibiotics have been inadvertently taken in the European region, according to a behavioral insight study by the World Health Organization (WHO) [49]. According to emerging data from interventional studies and animal models, the microbiota may be a key factor in the development of protective antibody responses to vaccination. For instance, mice that had been given antibiotics and kept germ-free exhibited lower antibody reactions to the seasonal influenza vaccine. Therefore, recognizing that the microbiota plays a crucial role in regulating immunological responses to vaccination, microbiota-targeted therapies are a promising strategy to maximize the effectiveness of the COVID-19 vaccine in addition to the treatment with COVID-19 treatment [50]. Gut dysbiosis appears to be a common clinical feature in patients with COVID-19. COVID-19 patients have a threefold increase in the diversity of fungi and pathogens. More severe symptoms were associated with patients with a high abundance of more than two *Aspergillus* pathogens [23, 51]. Antibiotic therapy can decrease the diversity of gut microbiota species, stimulate the development of resistance to bacterial antibiotics, and disturb the balance that usually exists, causing bacterial overgrowth such as toxigenic *C. difficile*. In adults, a study has shown that a combination use of meropenem, gentamicin, and vancomycin decreased the prevalence of bifidobacterium and butyrate-producing species. Furthermore, the study has revealed that the baseline composition of the intestinal microbiota was mostly restored in 1.5 months, while many other common species remained undetectable [52, 53]. As a result, antibiotic resistance has become an important public health issue worldwide [54].

The impact of patient nutrition and probiotic therapy on COVID-19 is another topic of interest. Many approaches have been studied to modulate and improve the intestinal microbiota in patients with COVID-19 by supplementation with probiotics, prebiotics, trace elements, and bacterial metabolites, revealing that supplementation could reduce the hyperinflammatory response and severity of COVID-19 [55]. Several studies explored the impact of the intestinal microbiota on respiratory infections and systemic immunity. They revealed the essential role of the commensal microbiota in enhancing antiviral responses through modulation of immune responses during normal conditions and viral infections, especially in the respiratory tract [56, 57]. Furthermore, a higher mortality rate in respiratory infections is associated with intestinal dysbiosis, possibly due to decreased secretion of regulatory immune cells (T cells) in GIT and respiratory [58]. Studies were carried out on COVID-19 patients, as patients may have unbalanced microbiota in the pharynx in addition to GIT and lungs, reinforcing the idea of a gut–lung axis where any disturbances in the mucosa of GIT may have other sites [59–61]. Human microbiota and probiotics can increase health benefits due to immunomodulatory effects, IgA secretion, and the activity of neutrophils and macrophages. Consequently, probiotics can protect the host against viral infections and many respiratory viruses, such as COVID-19, and prevent secondary bacterial infections [62].

However, COVID-19 can develop microbiota dysbiosis in the lungs and gut 6 months after recovery [63]. Therefore, decreased intestinal microbiomes, such as lactobacillus and bifidobacterium, and poor nutrition intake can hinder recovery [64]. Therefore, probiotics supplements have shown a significant effect in improving COVID-19 symptoms of COVID-19 such as diarrhea, headache, and cough, and support the microbiota balance in the intestine–lung axis [65, 66].

### Strengths and limitations

This bibliometric study examines the links between COVID-19 and microbiota research. This study employs a well-known scientometric software tool in the construction and visualization of bibliometric networks (VOSviewer). However, there are some limitations that come with this study. To begin, our investigation is predicated mostly on quantitative analysis and somewhat marginally on qualitative analysis. Second, the retrieval is carried out primarily through the use of the Scopus database. However, it is important to note that Scopus is the most widely used scientometrics database and that visualization-based literature analysis lays the groundwork for scholars to grasp the hotspots and trends in this subject easily.

## Conclusions

This study provides an overall picture of the links between microbiota and COVID-19. Hotspots in this field were “COVID-19 is associated with an altered upper respiratory tract microbiome,” “the effect of antibiotics on the gut microbiome,” as well as “patient nutrition and probiotic therapy in COVID-19.” This landscape analysis of the links between the microbiota and COVID-19 will provide a basis for future research.

## Data Availability

The datasets generated and/or analyzed during the current study are available upon request from the corresponding author.

## Abbreviations

SARS‑CoV‑2: Severe acute respiratory syndrome coronavirus 2
COVID-19: Coronavirus disease 2019
CIT: Gastrointestinal tract
